# Prospective Association of the Mediterranean Diet with the Onset of Cardiometabolic Multimorbidity in a UK-Based Cohort: The EPIC-Norfolk Study

**DOI:** 10.1016/j.tjnut.2024.10.027

**Published:** 2024-10-17

**Authors:** Qiaoye Wang, Amand Floriaan Schmidt, S Goya Wannamethee

**Affiliations:** 1 Department of Primary Care and Population Health, Institute of Epidemiology and Health Care, University College London, London, United Kingdom; 2 Department of Population Science and Experimental Medicine, Institute of Cardiovascular Science, University College London, London, United Kingdom; 3 Department of Cardiology, Amsterdam Cardiovascular Sciences, Amsterdam University Medical Centre, University of Amsterdam, Amsterdam, The Netherlands

**Keywords:** Mediterranean diet, cardiometabolic health, cardiometabolic disease, multimorbidity, cardiometabolic multimorbidity

## Abstract

**Background:**

Cardiometabolic multimorbidity (CMM), defined as the co-occurrence of 2 or more cardiometabolic diseases, including myocardial infarction (MI), stroke, and type 2 diabetes (T2D), is an increasing public health challenge. Although poor diet is a known risk factor for a first cardiometabolic disease (FCMD), the relationship with subsequent occurrence of CMM is less studied.

**Objectives:**

This study aims to investigate the prospective association between baseline adherence to the Mediterranean diet and the onset of CMM across various follow-up durations.

**Methods:**

We used data from the European Prospective Investigation into Cancer-Norfolk cohort study of 21,900 adults, aged 40–79 free of prevalent MI, stroke, and T2D at baseline (1993–1997). A median-based Mediterranean diet score and a pyramid-based MDS (pyr-MDS) were used to measure baseline adherence to the Mediterranean diet. Multistate modeling was employed to investigate associations with the FCMD and the subsequent CMM event.

**Results:**

Over the entire follow-up period of 21.4 y (median), we observed 5028 FCMD and 734 CMM events. Multistate analysis indicated that the association between baseline Mediterranean diet and the risk of CMM may be stronger in shorter follow-up durations. Particularly, baseline pyr-MDS was significantly associated with the risk of subsequent CMM transitioning from FCMD when follow-up durations were limited to 10 and 15 y, with hazard ratio (95% confidence interval) being 0.67 (0.53, 0.84) and 0.80 (0.70, 0.92) per SD increase in pyr-MDS, respectively. Additionally, we observed that the risk of CMM transitioning from FCMD was modified by social class across shorter to longer follow-ups, where the impact of baseline Mediterranean diet was only significant in nonmanual workers.

**Conclusions:**

Baseline adherence to the Mediterranean diet was potentially associated with a lower risk of CMM transitioning from FCMD, particularly during shorter follow-up periods.

## Introduction

With the worldwide population aging and decreased mortality from major chronic diseases, it has become increasingly common for people to have several co-occurring diseases, known as multimorbidity [1]. Multimorbidity would not only significantly impact the affected individual’s wellbeing and life quality, but also burden healthcare systems [[1], [2], [3]]. Cardiometabolic diseases (CMD), including myocardial infarction (MI), stroke, and type 2 diabetes (T2D), are a group of diseases that have shared risk factors and tend to cluster together [3,4]. With their inter-related physiological pathways, the coexistence of 2 or more CMD, referred to as cardiometabolic multimorbidity (CMM), is 1 of the most common patterns of multimorbidity [4,5]. The prevalence of CMM was estimated to be around 3%–6% in the United Kingdom populations [6,7]. CMM has been shown to be associated with an increased risk of mortality: individuals aged 60 with 2-disease CMM and 3-disease CMM were estimated to have 12 y and 15 y of reduction in life expectancy [3].

Poor diet is a well-identified modifiable risk factor for CMD [8]. The Mediterranean diet, characterized by higher intakes of plant-based foods, fish, and olive oil, and lower intakes of meat and dairy products, is 1 of the most well-examined healthy dietary patterns [9]. Substantial evidence supports the inverse association between adhering to the Mediterranean diet and risk of CMD [[10], [11], [12], [13], [14]]. Particularly, previous reports from the European Prospective Investigation into Cancer (EPIC) studies have shown the inverse association of pyramid-based or relative Mediterranean diet score (MDS) with the risk of ischemic heart disease, stroke, and T2D [hazard ratio (HR) (95%): 0.94 (0.90, 0.98), 0.93 (0.87, 0.99), and 0.88 (0.79, 0.97), respectively, per 1 standard deviation (SD) increase in the MDS] [13,14].

Although the Mediterranean diet was found to impact the development of a first CMD (FCMD), whether the beneficial effects of the Mediterranean diet would carry on to affect the risk of developing a subsequent CMD, which is CMM, remain less studied. One recent older British men cohort study investigated the association between baseline Mediterranean diet and CMM but did not find significant association [6]. However, this study was limited to males >60 y old, so its findings may not be generalizable to the broader United Kingdom population. Meanwhile, the previous study estimated the associations over a follow-up period of ∼20 y, which may not capture the potential effect attenuations between baseline Mediterranean diet and the risk of CMM with continuing follow-up. Besides, this prior study assessed Mediterranean diet with an adapted MDS, Elderly Dietary Index (EDI), which was specifically developed for older adults. MDS developed on the basis of different dietary guidelines may impact their effectiveness in predicting CMD risks. The previous EPIC-Norfolk study has found that the pyramid-based MDS (pyr-MDS), computed according to the Mediterranean Diet Foundation proposal, was more strongly associated with and may be a better predictor of cardiovascular diseases (CVD), compared with the most frequently used median-based MDS (m-MDS) [13]. However, it remains unclear if the pyr-MDS retains a stronger association with the risk of CMM.

In the current study, we aim to investigate the prospective association of baseline adherence to the Mediterranean diet, using 2 MDS (that is, pyr-MDS and m-MDS), with the risk of CMM across various follow-up durations in a general United Kingdom population cohort – the EPIC-Norfolk study. Particularly, our study aims to explore how baseline Mediterranean diet adherence may impact the disease transition process of CMM, from free of CMD at baseline to FCMD, and subsequently to CMM, and how different follow-up durations may impact the associations.

## Methods

### Study population

EPIC-Norfolk is a large ongoing UK-based prospective cohort study started from 1993 to 1997. This cohort study recruited >30,000 men and women aged 40–79 y from eastern England through GP registers [15]. At baseline, participants completed a health and lifestyle questionnaire, as well as a food-frequency questionnaire (FFQ), and were invited to a health check. Morbidity and mortality were followed till March 2018. For the current study, we excluded participants who reported a history of MI, stroke, or diabetes, and those who were under diabetic medications at baseline (*n =* 2491). Ethical approval for the study was granted by the Norwich District Ethics Committee and participants gave informed consent (Rec Ref: 98NC01).

### Dietary assessment and Mediterranean diet scores (MDS)

A 130-item semiquantitative FFQ was used to obtain participants’ habitual diet at baseline. For each food item, participants were asked to report their average intake over the past year. Major foods and nutrients collected from the FFQ have been validated against 16-d weighted dietary records, 24-h dietary recall, and selected biomarkers in the subsample of EPIC-Norfolk [[16], [17], [18]]. Also, reproducibility of the dietary assessment has been assessed in a previous EPIC-Norfolk report [13].

Two MDS were derived on the basis of the FFQ. The first MDS was the most commonly used MDS in the literature, which assigned component scores on the basis of cohort sex-specific medians (m-MDS) [19]. The m-MDS had 9 dietary components, including vegetables, fruits and nuts, legumes, cereal, fish, meat, dairy, alcohol, and ratio of monounsaturated and polyunsaturated fatty acids to saturated fatty acids. For each dietary component, a score of 0 or 1 was assigned on the basis of the comparison to cohort sex-specific medians (Supplemental Table 1). The other MDS was a pyr-MDS developed on the basis of the Mediterranean Diet Foundation proposal, which accounted for both the traditional Mediterranean diet and the contemporary food environment and can be applied to both Mediterranean and non-Mediterranean regions [13,20]. The pyr-MDS included 15 dietary components, which were vegetables, legumes, fruits, nuts, cereals, dairy, fish, red meat, processed meat, white mat, egg, potato, sweets, alcohol, and olive oil. For each dietary component, a continuous score of 0–1 was assigned according to the participant’s degree of adherence to the recommendation (Supplemental Table 2). For the calculations of MDS, dietary intakes have been adjusted to a 2000 kcal/d diet using the residual method, so that diet quality assessed in the current study is independent of diet quantity, and measurement errors because of under- or over-reporting of dietary consumption can be partly reduced [13].

### Outcome ascertainment

Participants were followed for acute myocardial infarction (AMI), stroke, T2D, and death from baseline (1993–1997) to 31 March, 2018. Both fatal and nonfatal AMI and stroke were counted as diagnoses of CMD. Nonfatal CMD were identified from hospital admission records, and death information was obtained from the death certification at the Office for National Statistics [21]. Causes of death were coded by nosologists according to the International Classification of Diseases (ICD). Fatal AMI was coded using ICD9 410 or ICD10 I21, and fatal stroke was coded using ICD9 430-438 or ICD10 I60-I69 as underlying causes of death. CMM was ascertained at the date when the participant was diagnosed or died of a second type of CMD.

### Covariates

Sociodemographic and lifestyle characteristics, including age (years), sex (male or female, with female coded as 1), BMI (kg/m^2^), smoking status (current, former, and never), physical activity, social class, marital status, education level, current use of antihypertensive drugs or lipid-lowering drugs, and family history of MI, stroke, or diabetes, were collected at baseline through self-administered questionnaire. According to occupational physical activity and time spending in physical exercise, physical activity was categorized into 4 groups: physically inactive, moderately inactive, moderately active, and active [22]. Social class was classified into 2 groups on the basis of participants and their partners’ occupations: nonmanual workers, including professionals, managerial and technical occupations and nonmanual skilled workers, and manual workers, including manual skilled workers, semiskilled workers, and unskilled manual workers [23]. Marital status was grouped into 2 categories: married or other (including single, widowed, separated, or divorced). Educational status was based on the highest qualification attained and was categorized into 4 groups: bachelor’s degree or above, A-level or equivalent (that is, advanced level qualifications before entrance to university), O-level or equivalent (that is, ordinary-level qualifications before A-level), and less than O-level or no qualifications [24]. At baseline health check, each participant’s weight (kg) and height (cm) were measured by trained nurses.

### Statistical analyses

Baseline characteristics were grouped by adherence to the Mediterranean diet (quartiles of m-MDS and pyr-MDS). Continuous variables were summarized as means with SD, and categorical variables were summarized with frequency (percentage) across quartiles of the MDS. Kruskal–Wallis test was used to compare the difference of continuous variables, and Fisher’s exact test was conducted to compare the difference of categorical variables.

Cox’s proportional hazards regression model was first used to estimate HR and 95% confidence interval (CI) for associations between the MDS and risk of CMM. Each MDS was modeled continuously per SD and categorically (quartiles) with equal numbers of participants in each quartile. Analyses were adjusted for potential confounders: age, sex, BMI, smoking status, physical activity, social class, marital status, education level, medication use (antihypertensive drugs or lipid-lowering drugs), and family history of MI, stroke, or diabetes. The proportionality of hazards was graphically assessed by plotting scaled Schoenfeld residuals against time and found no significant trend with time. We conducted complete-case analysis in the current study, excluding individuals who had any missing demographic or dietary data (*n =* 5985), which left 21,900 participants in the analysis. To explore potential biases because of missing data, we also conducted a multiple imputation (*n =* 10) following Cox regression analysis. Estimates from 10 imputed datasets were pooled under Rubin’s rules.

**FIGURE 1:**
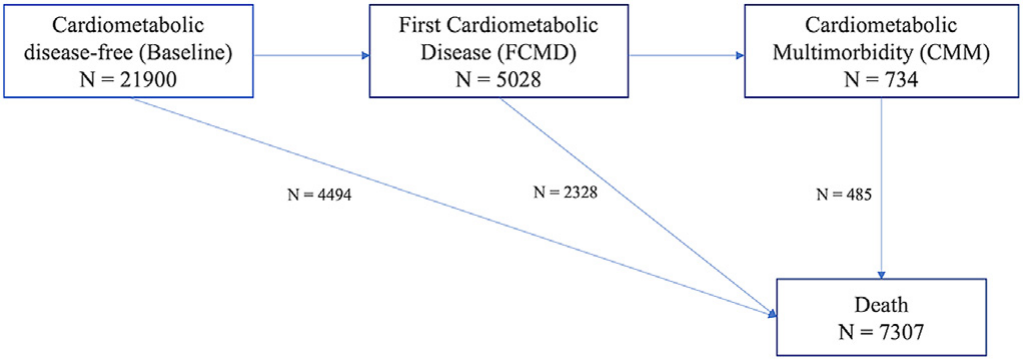
Disease transitions from baseline (free of cardiometabolic disease) to first cardiometabolic disease, subsequently to cardiometabolic multimorbidity, and death for EPIC-Norfolk study participants over a median follow-up of 21.4 y (*N =* 21,900).

After Cox regression analyses, we conducted multistate modeling to investigate the impact of baseline adherence to the Mediterranean diet on CMM disease transitions from baseline (free of CMD) to FCMD, and from FCMD to CMM, in the follow-up duration of 10 y, 15 y, and entire follow-up period [25]. Death was included in the disease transitions as the competing risk and final absorbing state. Specifically, for each MDS, 2 multistate models were conducted to assess the CMM disease transition risks. The first multistate model assessed the CMM disease transition risks without differentiating the FCMD, estimating the risk of transitioning from baseline to FCMD, and from FCMD to CMM (Figure 1). In the second model (Supplemental Figure 1), FCMD were differentiated into individual diseases (that is, first AMI, stroke, or T2D), allowing for separate estimations of transition risks from baseline to first AMI, stroke, or T2D, and from first AMI, stroke, or T2D to CMM. Potential nonlinear relationships were examined using the restricted cubic splines. Moreover, because BMI could potentially be mediating the relationship of Mediterranean diet and transition risk of CMM, to test the robustness of the multistate model results, we investigated the transition risks excluding BMI from the model. Lastly, to explore the presence of effect modification on the association of Mediterranean diet with the transition risk of CMM, we also conducted subgroup analyses according to age (<60 or ≥60 y old), sex, social class (nonmanual or manual workers), and adherence to the Mediterranean diet (low or high by median pyr-MDS).

Results are presented as adjusted HRs with 95% CIs. All the analyses were conducted using R (version 4.2.2), leveraging the packages: survival (version 3.4.0), mice (version 3.15.0), and mstate (version 0.3.2).

## Results

### Baseline characteristics of participants

Of 21,900 study participants free of any CMD at baseline, during the entire follow-up period, with a median follow-up of 21.4 y (Q1, Q3: 18.8, 22.9), 5028 individuals developed ≥1 CMD. A total of 734 participants progressed to CMM following the FCMD (Figure 1).

Table 1 presents the baseline characteristics of EPIC-Norfolk study participants by their Mediterranean diet adherence in 1993–1997. Comparing with participants with lower adherence to the Mediterranean diet at baseline, people with higher adherence were younger, more likely to have a college education, and working as nonmanual workers, less likely to be current smokers or being physically inactive, but more likely to have a family history of MI and under lipid-lowering medications. The 2 MDS, though derived on the basis of different scoring system, were moderately correlated, with Spearman’s correlation 0.53 (*P* value < 0.01). Participants with missing data had higher incident CMM rate but similar MDS comparing to complete samples (Supplemental Table 3).

**TABLE 1:** Cohort characteristics of 21,900 EPIC-Norfolk study participants aged 40–79 in 1993–1997, according to baseline adherence to the Mediterranean diet

Characteristics	Baseline MDS							
	m-MDS				pyr-MDS			
Quartile (score)	**Q1** (0–3)	**Q2** (4)	**Q3** (5)	**Q4** (6–9)	**Q1** (0–7.55)	**Q2** (7.56–8.45)	**Q3** (8.46–9.34)	**Q4** (9.35–15)
Number of participants, *n*	7277	4512	4317	5794	5479	5502	5465	5454
Male, *n* (%)	3234 (44.4)	1954 (43.3)	1860 (43.1)	2583 (44.6)	3191 (58.2)^1^	2602 (47.3)	2196 (40.2)	1642 (30.1)
Age (y)	58.9 (9.3)^1^	58.8 (9.3)	58.6 (9.2)	58.1 (9.1)	59.0 (9.3)^1^	59.2 (9.3)	58.5 (9.2)	57.7 (8.9)
BMI (kg/m^2^)	26.2 (3.9)	26.2 (3.8)	26.3 (3.9)	26.2 (3.8)	26.3 (3.7)^1^	26.3 (3.9)	26.3 (3.9)	26.0 (3.9)
Education level, *n* (%)								
Bachelor’s degree or above	722 (9.9)^1^	564 (12.5)	597 (13.8)	1007 (17.4)	414 (7.6)^1^	615 (11.2)	747 (13.7)	1114 (20.4)
Marital status, *n* (%)								
Married	5926 (81.4)^1^	3700 (82.0)	3576 (82.8)	4865 (84.0)	4670 (85.2)^1^	4620 (84.0)	4471 (81.8)	4306 (79.0)
Smoking status, *n* (%)								
Current smokers	1205 (16.6)^1^	529 (11.7)	403 (9.3)	423 (7.3)	966 (17.6)^1^	651 (11.8)	550 (10.1)	393 (7.2)
Physical activity level, *n*(%)								
Inactive	2347 (32.3)^1^	1353 (30.0)	1193 (27.6)	1369 (23.6)	1720 (31.4)^1^	1687 (30.7)	1566 (28.7)	1289 (23.6)
Social class, *n*(%)								
Nonmanual workers	4042 (55.5)^1^	2718 (60.2)	2675 (62.0)	3846 (66.4)	2773 (50.6)^1^	3145 (57.2)	3454 (63.2)	3909 (71.7)
Family history of MI, *n* (%)	2489 (34.2)^1^	1642 (36.4)	1547 (35.8)	2259 (39.0)	1863 (34.0)^1^	1962 (35.7)	1973 (36.1)	2139 (39.2)
Family history of stroke, *n* (%)	1783 (24.5)	1100 (24.4)	1040 (24.1)	1412 (24.4)	1316 (24.0)	1324 (24.1)	1348 (24.7)	1347 (24.7)
Family history of diabetes, *n*(%)	899 (12.4)	569 (12.6)	527 (12.2)	757 (13.1)	721 (13.2)	670 (12.2)	701 (12.8)	660 (12.1)
Use of antihypertensive drug, *n*(%)	1124 (15.5)	709 (15.7)	688 (15.9)	958 (16.5)	833 (15.2)^1^	923 (16.8)	896 (16.4)	827 (15.2)
Use of lipid-lowering drugs, *n*(%)	38 (0.5)^1^	41 (0.9)	44 (1.0)	104 (1.8)	30 (0.6)^1^	64 (1.2)	66 (1.2)	67 (1.2)

Abbreviations: m-MDS, median-based Mediterranean diet score; CMM: Cardiometabolic multimorbidity; pyr-MDS, pyramid-based Mediterranean diet score; SD, standard deviation.

Summary statistics in mean (SD) or frequency (percentage) unless stated otherwise.

Fisher’s exact test was used for all categorical variables.

Kruskal–Wallis test was used for all continuous variables.

1 *P* value < 0.05

### Mediterranean diet and risk of cardiometabolic multimorbidity

**FIGURE 2:**
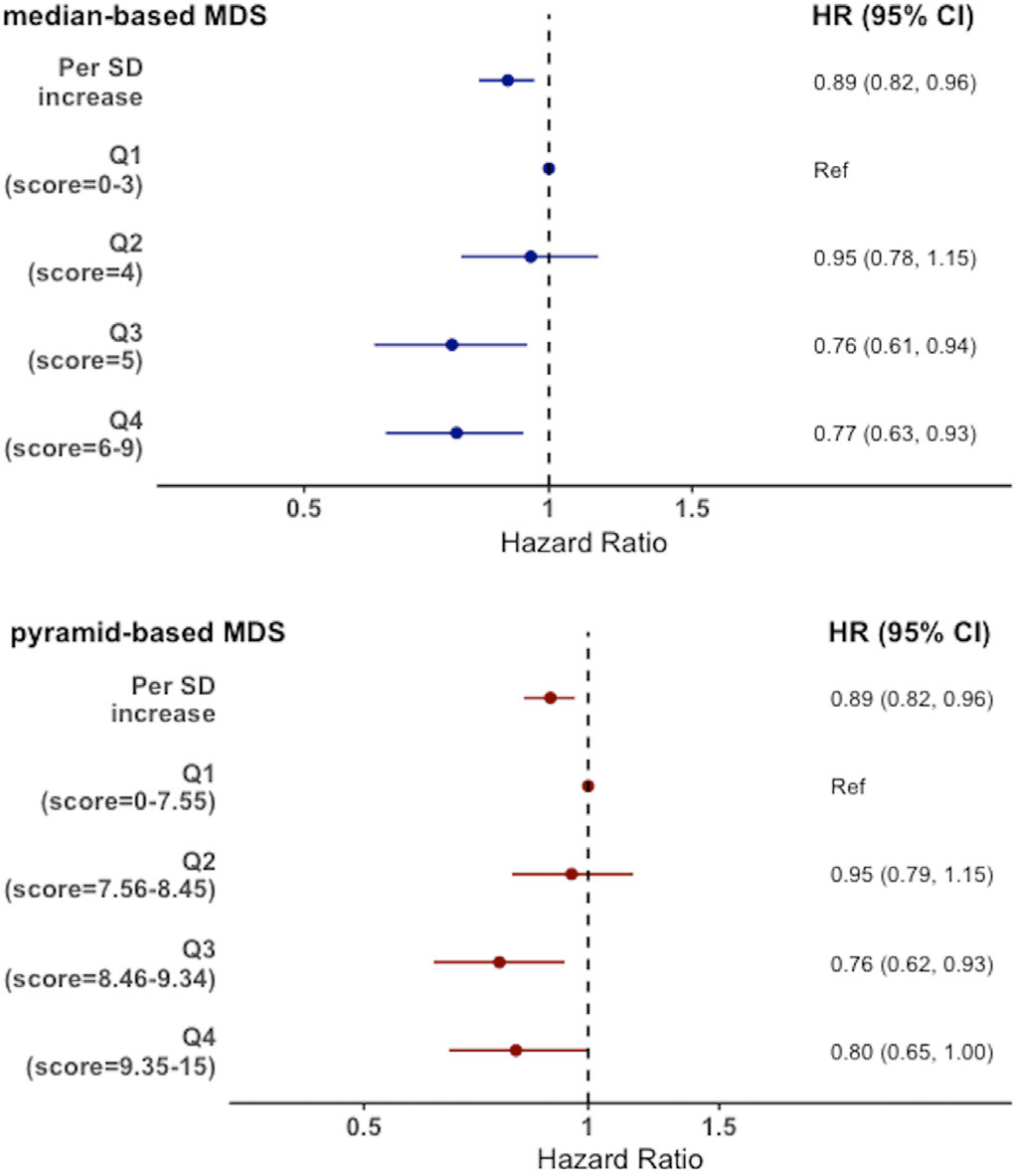
Prospective association between baseline adherence to the Mediterranean diet and the risk of cardiometabolic multimorbidity in EPIC-Norfolk participants aged 40–79 in 1993–1997, over a median follow-up of 21.4 y (*N =* 21,900). CI, Confidence interval; HR, hazard ratio; MDS, Mediterranean diet score; Ref, reference group; SD, standard deviation. Each SD unit corresponds to 1.78 m-MDS and 1.32 pyr-MDS points. Model adjusted for age, sex, BMI, smoking status, physical activity, social class, marital status, education level, medication use (antihypertensive drugs or lipid-lowering drugs), and family history of MI, stroke, or diabetes.

Cox regression analyses showed that baseline adherence to the Mediterranean diet was significantly associated with a lower risk of CMM over a median follow-up of 21.4 y (Figure 2). For each SD (1.78 point) increase in the m-MDS, the HR (95% CI) of CMM was 0.89 (0.82, 0.96) after adjusting for sociodemographic and lifestyle covariates. Pyr-MDS had the same estimate of 0.89 (0.82, 0.96) per 1 SD (1.32 point) increase. The results did not change substantially after imputing missing data (Supplemental Table 4).

### Mediterranean diet and disease transition risks of cardiometabolic multimorbidity

TABLE 2, TABLE 3 present the associations between baseline Mediterranean diet and CMM disease transition risks across various follow-up durations. The results suggested that greater adherence to the Mediterranean diet at baseline was associated with a lower risk of transitioning from baseline to FCMD, and with a lower risk of CMM transitioning from FCMD, particularly in the follow-up durations of 10 and 15 y for pyr-MDS [HR (95% CI) of CMM from FCMD: 0.67 (0.53, 0.84) and 0.80 (0.70, 0.92) per SD increase in pyr-MDS for 10-y and 15-y follow-ups, respectively]. However, over the entire follow-up period of >20 y, baseline adherence to the Mediterranean diet was associated only with the risk of FCMD, but not statistically significantly with the risk of CMM transitioning from FCMD. The analyses of individual FCMD suggested that adherence to the Mediterranean diet, as measured by the pyr-MDS, may be particularly associated with the risk of CMM transitioning from first AMI and T2D, over the10-y and 15-y follow-up periods (Supplemental Tables 5–6).

**TABLE 2:** HRs (95% CI) for disease transitions from baseline (CMD-free) to FCMD and CMM by m-MDS in EPIC-Norfolk study participants aged 40–79 y in 1993–1997, over the follow-up duration of 10, 15, and a median of 21.4 y (*N =* 21,900)

Follow-up duration of 10 (y)		HRs (95% CI) for each disease transition				
		Baseline m-MDS				
Disease transition	No. of Events	Per SD increase	Q1	Q2	Q3	Q4
Baseline to FCMD	1357	0.94 (0.89, 0.99)^1^	Ref	0.95 (0.82, 1.10)	0.85 (0.73, 1.00)^1^	0.87 (0.76, 1.01)
FCMD to CMM	97	0.92 (0.75, 1.14)	Ref	1.17 (0.69, 1.99)	0.97 (0.54, 1.74)	0.77 (0.42, 1.40)

**Follow-up duration of 15 (y)**		**HRs (95% CI) for each disease transition**				
		**Baseline m-MDS**				

**Disease transition**	**No. of events**	**Per SD increase**	**Q1**	**Q2**	**Q3**	**Q4**

Baseline to FCMD	2674	0.92 (0.89, 0.96)^1^	Ref	0.96 (0.87, 1.07)	0.90 (0.81, 1.00)	0.84 (0.76, 0.94)^1^
FCMD to CMM	260	0.91 (0.80, 1.03)	Ref	1.12 (0.82, 1.55)	0.89 (0.63, 1.27)	0.82 (0.58, 1.15)

**Entire follow-up duration - a median of 21.4 y**		**HRs (95% CI) for each disease transition**				
		**Baseline m-MDS**				
**Disease transition**	**No. of Events**	**Per SD increase**	**Q1**	**Q2**	**Q3**	**Q4**

Baseline to FCMD	5028	0.93 (0.90, 0.95)^1^	Ref	0.93 (0.86, 1.01)	0.90 (0.83, 0.97)^1^	0.85 (0.79, 0.92)^1^
FCMD to CMM	734	0.94 (0.87, 1.01)	Ref	1.04 (0.86, 1.27)	0.80 (0.64, 0.99)^1^	0.86 (0.70, 1.05)

Abbreviations: CI, confidence interval; CMM, cardiometabolic multimorbidity; FCMD, first cardiometabolic disease; HR, hazard ratio; m-MDS, median-based Mediterranean diet score; SD, standard deviation.

Each SD unit corresponds to 1.78 m-MDS points. m-MDS Q1: score = 0–3, *n =* 7277; Q2: score = 4, *n =* 4512; Q3: score = 5, *n =* 4317; Q4: score = 6–9, *n =* 5794. Model adjusted for age, sex, BMI, smoking status, physical activity, social class, marital status, education level, medication use (antihypertensive drugs or lipid-lowering drugs), and family history of MI, stroke, or diabetes.

^1^*P* value < 0.05

**TABLE 3:** HRs (95% CI) for disease transitions from baseline (CMD-free) to FCMD and CMM by pyr-MDS in EPIC-Norfolk study participants aged 40–79 y in 1993–1997, over the follow-up duration of 10, 15, and a median of 21.4 y (*N =* 21,900)

Follow-up duration of 10 y		HRs (95% CI) for each disease transition				
		Baseline pyr-MDS				
Disease transition	No. of Events	Per SD increase	Q1	Q2	Q3	Q4
Baseline to FCMD	1357	0.91 (0.86, 0.96)^1^	Ref	0.85 (0.74, 0.98)^1^	0.89 (0.77, 1.03)	0.75 (0.64, .89)^1^
FCMD to CMM	97	0.67 (0.53, 0.84)^1^	Ref	0.60 (0.36, 1.02)	0.44 (0.24, 0.80)^1^	0.47 (0.25, 0.90)^1^

**Follow-up duration of 15 y**		**HRs (95% CI) for each disease transition**				
		**Baseline pyr-MDS**				
**Disease transition**	**No. of Events**	**Per SD increase**	**Q1**	**Q2**	**Q3**	**Q4**

Baseline to FCMD	2674	0.91 (0.87, 0.94)^1^	Ref	0.88 (0.79, 0.97)^1^	0.86 (0.77, 0.96)^1^	0.77 (0.69, 0.87)^1^
FCMD to CMM	260	0.80 (0.70, 0.92)^1^	Ref	0.88 (0.65, 1.20)	0.55 (0.38, 0.80)^1^	0.74 (0.52, 1.06)

**Entire follow-up duration-a median of 21.4 y**		**HRs (95% CI) for each disease transition**				
		**Baseline pyr-MDS**				
**Disease transition**	**No. of events**	**Per SD increase**	**Q1**	**Q2**	**Q3**	**Q4**

**Baseline to FCMD**	5028	0.93 (0.90, 0.95)^1^	Ref	0.92 (0.86, 1.00)	0.88 (0.81, 0.95)^1^	0.83 (0.76, 0.90)^1^
**FCMD to CMM**	734	0.94 (0.87, 1.01)	Ref	1.03 (0.85, 1.25)	0.83 (0.68, 1.02)	0.91 (0.74, 1.13)

Abbreviations: CI, confidence interval; CMM, cardiometabolic multimorbidity; FCMD, first cardiometabolic disease; HR, hazard ratio; pyr-MDS, pyramid-based Mediterranean diet score.

Each SD unit corresponds to 1.32 pyr-MDS points. pyr-MDS Q1: score = 0–7.55, *n =* 5479; Q2: score = 7.56–8.45, *n =* 5502; Q3: score = 8.46–9.34, *n =* 5465; Q4: score = 9.35–15, *n =* 5454.

Model adjusted for age, sex, BMI, smoking status, physical activity, social class, marital status, education level, medication use (antihypertensive drugs or lipid-lowering drugs), and family history of MI, stroke, or diabetes.

^1^*P* value < 0.05

Analyses using restricted cubic splines did not support potential nonlinear associations between Mediterranean diet (m-MDS and pyr-MDS) and CMM (Supplemental Figure 2). And excluding BMI from the model had similar estimates and findings (Supplemental Table 7). Stratified analysis showed significant effect modification by social class on the association between pyr-MDS and the CMM risk over the median follow-up of 21.4 y (*P* value for interaction = 0.01). Table 4 presents the estimated subgroup associations in manual and nonmanual workers. For nonmanual workers, baseline adherence to the Mediterranean diet was not only related to a lower risk of FCMD, but also associated with a lower risk of CMM transitioning from FCMD [0.89 (95% CI: 0.80, 0.98) per SD increase in pyr-MDS]. But for manual workers, Mediterranean diet adherence did not associate with the transition risk of CMM from FCMD [1.01 (95% CI: 0.90, 1.14) per SD increase in pyr-MDS]. When limiting to 10- or 15 y of follow-up durations, the significant effect modification of social class remains (*P* value for interaction <0.01). The impacts of the Mediterranean diet were consistent across age and sex groups (Supplemental Tables 8–9). Also, the effects of MDS did not differ according to low or high adherence to the Mediterranean diet (Supplemental Table 10).

**TABLE 4:** HRs (95% CI) for disease transitions from baseline (CMD-free) to FCMD and CMM by pyr-MDS in EPIC-Norfolk study aged 40–79 y in 1993–1997 over a median follow-up of 21.4 y, stratified by social class (*N =* 21,900)

		HRs (95% CI) for each disease transition				
**Nonmanual workers (*n* = 13,281)**		**Baseline pyr-MDS**				
**Disease transition**	**No. of events**	**Per SD increase**	**Q1**	**Q2**	**Q3**	**Q4**

Baseline to FCMD	2885	0.94 (0.90, 0.98)^1^	Ref	0.93 (0.84, 1.03)	0.90 (0.81, 1.00)	0.84 (0.76, 0.94)^1^

FCMD to CMM	405	0.89 (0.80, 0.98)^1^	Ref	0.91 (0.70, 1.18)	0.74 (0.56, 0.97) 1	0.77 (0.58, 1.03)

**Manual workers (*n* = 8619)**		**Baseline pyr-MDS**				
**Disease transition**	**No. of events**	**Per SD increase**	**Q1**	**Q2**	**Q3**	**Q4**

Baseline to FCMD	2143	0.91 (0.87, 0.95)^1^	Ref	0.92 (0.83, 1.03)	0.85 (0.76, 0.96)^1^	0.82 (0.72, 0.94)^1^

FCMD to CMM	329	1.01 (0.90, 1.14)	Ref	1.16 (0.88, 1.52)	0.94 (0.69, 1.28)	1.12 (0.80, 1.58)

*P* value for interaction = 0.01.

Abbreviations: CMM, cardiometabolic multimorbidity; FCMD, first cardiometabolic disease; HR, hazard ratio; pyr-MDS, pyramid-based Mediterranean diet score; Ref, reference group; SD, standard deviation.

Each SD unit corresponds to 1.32 pyr-MDS points. pyr-MDS Q1: score = 0–7.55, *n* for nonmanual worker = 2773, *n* for manual worker = 2706; Q2: score = 7.56–8.45, *n* for nonmanual worker = 3145, *n* for manual worker = 2357; Q3: score = 8.46–9.34, *n* for nonmanual worker = 3454, *n* for manual worker = 2011; Q4: score = 9.35–15, *n* for nonmanual worker = 3909, *n* for manual worker = 1545.

Model adjusted for age, sex, BMI, smoking status, physical activity, marital status, education level, medication use (antihypertensive drugs or lipid-lowering drugs), and family history of MI, stroke, or diabetes.

1 *P* value < 0.05

## Discussion

The current study investigated how baseline Mediterranean diet impacted the onset of CMM in the general United Kingdom population across various follow-up durations. We found that baseline Mediterranean diet was potentially related to a lower risk of CMM transitioning from FCMD in shorter follow-up periods.

### Comparison with other studies

The current study is the first to show how baseline adherence to the Mediterranean diet impact the disease transition of CMM in general United Kingdom population. To the best of our knowledge, only 3 prior studies conducted in the United Kingdom and China estimated the prospective association of diet with CMM. The Whitehall II cohort followed 8270 middle-aged British participants over a mean of 23.7 y [7]. The study measured dietary intake with the consumption of fruit and vegetables. It found that low consumption of fruit and vegetables was related to a higher risk of FCMD [HR (95% CI): 1.09 (1.00, 1.18)], but not associated with the transition risk of CMM from FCMD. Another study followed 0.5 million Chinese adults for a median duration of 11.2 y [26]. The study revealed that a less healthy dietary habit, defined as nondaily intakes of vegetables, fruits, and eggs, or eating red meat daily or less than weekly, was significantly associated with a higher risk of FCMD, specifically first ischemic stroke [HR (95% CI): 1.23 (1.14, 1.33)]. For the disease transition of CMM from FCMD, the study demonstrated that an unhealthy diet was associated with the transition risk of CMM only when ischemic stroke or hemorrhagic stroke was the FCMD [HR (95% CI) of CMM: 1.25 (1.05, 1.49) and 1.84 (1.04, 3.25) for ischemic stroke and hemorrhagic stroke, respectively], but not from other FCMD. These 2 studies, however, did not measure the overall diet quality on the basis of any established healthy dietary pattern. The most recent study measured baseline diet with a Mediterranean diet-adapted dietary score EDI and investigated the impact of EDI on the onset of CMM in an older United Kingdom male cohort [that is, the British Regional Heart Study (BRHS) cohort] [6]. The study did not find significant association between baseline EDI and risk of FCMD or the risk of CMM transitioning from FCMD over a median follow-up of 19.3 y. This study, however, used a dietary score (EDI) that was developed particularly for older adults and did not include women or younger populations in the analyses. Additionally, the 2 previous cohorts of British adults both had lengthy follow-up durations (23.7 y in the Whitehall II study and 19.3 y in the BRHS), which may also impact the observed association between baseline diet and the risk of CMM [6,7].

### Interpretation of results and implications

Our analysis furthered previous studies by investigating the association of Mediterranean diet with CMM using 2 validated MDS in a large United Kingdom population cohort, including both men and women from a wide age range, and investigated the potential impacts of various follow-up durations. Our results suggested that the associations between baseline adherence to the Mediterranean diet and the risk of subsequent CMM following FCMD were more pronounced in shorter follow-up durations of 10 and 15 y.

There has been well-documented evidence on the primary preventive effects of the Mediterranean diet on CMD in healthy populations [27]. However, for patients with established CMD, the potential secondary preventive effects of the Mediterranean diet remain less studied. A few studies suggested that adhering to the Mediterranean diet may be associated with lower risk of another CMD for patients with existing CVD. For example, an Italian prospective cohort study of 8291 patients with recent MI found that a higher MDS was related to a significant lower risk of T2D after a mean follow-up of 3.2 y [HR (95% CI): 0.65 (0.49, 0.85)] [28]. For patients with T2D, most studies suggested significant benefits of adhering to the Mediterranean diet on the risk of CVD [29,30]. However, when separating individual CVD outcomes, a recent prospective cohort study of 22,473 diabetic participants from the United Kingdom Biobank did not observe significant impact of the Mediterranean diet particularly on MI or stroke risks [30].

Our study found an association between baseline adherence to the Mediterranean diet and the risk of FCMD across shorter to longer follow-up periods. Additionally, our study suggested that baseline adherence to the Mediterranean diet may be associated with the transition risks from FCMD, particularly from first AMI and T2D, to CMM. However, significant association with the risk of transitioning to CMM following FCMD was only observed in follow-up periods of 10 and 15 y. This finding indicated that CMM events occurred during longer follow-up may become less related to the baseline diet. Although no significant violations of the proportionality of hazards against time were found, there might be a modest nonproportional hazards effect that cumulatively affected the estimated transitioning risk of CMM following FCMD. Specifically, over time, the baseline dietary score may become less accurate in capturing participants’ diet quality, especially after the development of FCMD, thus weakening the observed association. Meanwhile, as the cohort ages over a long follow-up period, the impact of baseline Mediterranean diet may be attenuated. Although our study did not find significant effect modification by age, previous studies have indicated that the influence of modifiable cardiometabolic risk factors related to dietary intake, such as total cholesterol and blood pressure, could be diminished in the elderly [31,32]. This attenuation may primarily be because of physiological changes associated with aging and higher mortality rates among older adults [33]. Overall, findings of our study add to the evidence that baseline adherence to the Mediterranean is potentially of great significance in preventing CMM subsequent to FCMD, with the effect being more pronounced during shorter follow-up periods.

Moreover, our current study observed a significant effect modification by social class groups in the association between baseline Mediterranean diet and the risk of CMM transitioning from FCMD. Although the Mediterranean diet was associated with a significant lower risk of CMM transitioning from FCMD in nonmanual workers, the association was attenuated in manual workers. It is important to note that this does not imply that manual workers should not consume a healthy diet to potentially prevent CMM. Instead, this result may be because of several potential reasons. First, our study did not capture more detailed food components in the Mediterranean diet, for example, the specific types of food items consumed (for example, specific fruits, vegetables, or fish). Individuals in lower socioeconomic status (SES) were found to prioritize prices over health values when selecting foods, which may result in less variety and the selection of foods with lower nutritional values [34]. It is therefore possible that differences in food items and nutrient consumption may explain the observed differences in the associations between manual and nonmanual workers. Additionally, our findings indicated that there may be underlying social factors for this effect modification that warrant further investigation. The observed difference may be explained by the notion that social class was related to other risk factors that could influence the development of CMM, such as medication adherence, self-care and management of CMD, access to preventive medical care, and quality of healthcare after FCMD [35,36].

### Comparison of Mediterranean diet scores

In the current study, we used 2 MDS, m-MDS and pyr-MDS, to evaluate participants’ adherence to the Mediterranean diet. Our results showed that the 2 MDS were similar in predicting the risk of CMM over the long follow-up duration, but the pyr-MDS may be a better predictor of CMM in shorter follow-ups comparing to the m-MDS. This finding was consistent with the prior EPIC-Norfolk study with a shorter follow-up (a mean of 12.2 y), showing a stronger association of pyr-MDS with first occurrence of CVD compared with m-MDS [13]. As the study suggested, the pyr-MDS may be more accurate and better in predicting CVD because it accounted for the continuous property of dietary consumption and took the contemporary food environment into account, such that it distinguished processed and unprocessed meat and included sweet foods. The m-MDS, though being the most frequently used MDS in the literature, could be too crude and not sensitive enough to capture Mediterranean diet adherence in the contemporary non-Mediterranean countries.

### Strengths and limitations

One key strength of this study is that it included a large cohort with long follow-up time. Also, evaluation of 2 MDS clarified the potential impacts of applying different Mediterranean diet-based scores with varying dietary characteristics. As limitations, the current study derived MDS from one-time dietary measure at baseline, so we could not account for potential changes in diet throughout the study period. Prior EPIC-Norfolk report has shown that the pyr-MDS was moderately reproducible over a 3.7-y period (ρ = 0.60) [13], but this did not necessarily capture the potential dietary change after the development of FCMD. People may change their diets after developing FCMD. A prospective study suggested that people tend to make favorable dietary changes, leading to increased diet quality, after being diagnosed with diabetes [37]. If adherence to the Mediterranean diet benefits the development of CMM, the lack of repeated dietary information, particularly the inability to capture potential dietary improvement after FCMD, could bias the estimated association with the transition from FCMD to CMM toward null, leading to underestimated HR. Second, the current study only identified patients with AMI at baseline, which did not recognize those with chronic coronary syndromes. Patients with chronic coronary syndromes at baseline may have a less healthy dietary habit, and this could lead to overestimation of our effect estimates. Or patients with prevalent coronary diseases may have modified and adopted a healthier dietary habit, and this could lead to underestimation of our effect estimates. Third, our current study did not differentiate subtypes of stroke. Han et al. (2021) study showed that dietary intakes may have different impacts on disease transitions of CMM with different first stroke subtypes - the occurrence of hemorrhagic stroke as the FCMD may benefit more from a healthy dietary intake in preventing the development of CMM [26]. However, >80% first stroke cases in the United Kingdom were found to be ischemic [38]. This may attenuate our estimated association of Mediterranean diet with the risk of CMM transitioning from FCMD. In addition, we could not rule out the effects of possible residual confounding, such as imprecise measurement of self-reported potential confounders or unmeasured confounders. Generalizability is limited because the current cohort is largely made of individuals from European ethnic origin. Minority ethnic groups in the United Kingdom were found to have higher CMD rates compared with the White population. Also, these groups exhibit different dietary habits from White individuals [39]. Our study, however, is unable to explore how diet may affect the risk of CMM in minority ethnic groups.

In conclusion, we observed a lower risk of CMM with higher baseline adherence to the Mediterranean diet in the current United Kingdom cohort. Our study suggests that, in shorter follow-up periods, adhering to the Mediterranean diet at baseline may be associated with lower risks of both FCMD and the subsequent CMM. Additionally, social class is a significant modifier of the association between the Mediterranean diet and risk of CMM transitioning from FCMD. The observed difference between nonmanual and manual workers warrants further investigation. Although the 2 MDS investigated in the current study demonstrate similar estimated associations with CMM over the long term, the pyr-MDS may be a better predictor of CMM in shorter follow-ups. Overall, findings of our study further highlight the importance of baseline adherence to the Mediterranean diet as it may benefit cardiometabolic health and potentially help prevent CMM in the general United Kingdom population.

## Funding

AFS was supported by BHF grant PG/22/10989, the UCL BHF Research accelerator AA/18/6/34223. The EPIC-Norfolk study (DOI 10.22025/2019.10.105.00004) has received funding from the Medical Research Council (MR/N003284/1 MC-UU_12015/1 and MC_UU_00006/1) and Cancer Research United Kingdom (C864/A14136). The genetics work in the EPIC-Norfolk study was funded by the Medical Research Council (MC_PC_13048). We are grateful to all the participants who have been part of the project and to the many members of the study teams at the University of Cambridge who have enabled this research.

## Data availability

Because of participant confidentiality and privacy concerns, data cannot be shared publicly and requests to access EPIC-Norfolk study data must be submitted in writing. Further information including the procedures to obtain and access data are described at https://www.epic-norfolk.org.uk/for-researchers/data-sharing/.

## Author contributions

The authors’ responsibilities were as follows – QW, SGW: contributed to the research conception and design; QW: performed the statistical analyses and prepared the first draft of the manuscript; AFS, SGW: commented and edited the previous versions of the manuscript; and all authors critically reviewed and approved the final manuscript.

## Conflict of interest

The authors report no conflicts of interest.

## References

[1] Tinetti M.E., Fried T.R., Boyd C.M. (2012). Designing health care for the most common chronic condition--multimorbidity. JAMA.

[2] Boyd C.M., Fortin M. (2010). Future of multimorbidity research: how should understanding of multimorbidity inform health system design?. Public Health Rev.

[3] Di Angelantonio E., Kaptoge S., Wormser D., Willeit P., Butterworth A.S., Bansal N. (2015). Association of cardiometabolic multimorbidity with mortality. JAMA.

[4] Busija L., Lim K., Szoeke C., Sanders K.M., McCabe M.P. (2019). Do replicable profiles of multimorbidity exist? systematic review and synthesis. Eur. J. Epidemiol..

[5] Prados-Torres A., Calderón-Larrañaga A., Hancco-Saavedra J., Poblador-Plou B., van den Akker M. (2014). Multimorbidity patterns: a systematic review. J. Clin. Epidemiol..

[6] Wang Q., Schmidt A.F., Lennon L.T., Papacosta O., Whincup P.H., Wannamethee S.G. (2023). Prospective associations between diet quality, dietary components, and risk of cardiometabolic multimorbidity in older British men. Eur. J. Nutr..

[7] Singh-Manoux A., Fayosse A., Sabia S., Tabak A., Shipley M., Dugravot A. (2018). Clinical, socioeconomic, and behavioural factors at age 50 y and risk of cardiometabolic multimorbidity and mortality: a cohort study. PLOS Med.

[8] Mozaffarian D. (2016). Dietary and policy priorities for cardiovascular disease, diabetes, and obesity: a comprehensive review. Circulation.

[9] Richardson L.A., Izuora K., Basu A. (2022). Mediterranean diet and its association with cardiovascular disease risk factors: a scoping review. Int J Environ Res Public Health.

[10] Guasch-Ferré M., Willett W.C. (2021). The mediterranean diet and health: a comprehensive overview. J. Intern. Med..

[11] Rosato V., Temple N.J., La Vecchia C., Castellan G., Tavani A., Guercio V. (2019). Mediterranean diet and cardiovascular disease: a systematic review and meta-analysis of observational studies. Eur. J. Nutr..

[12] Zeraattalab-Motlagh S., Jayedi A., Shab-Bidar S. (2022). Mediterranean dietary pattern and the risk of type 2 diabetes: a systematic review and dose–response meta-analysis of prospective cohort studies. Eur. J. Nutr..

[13] Tong T.Y.N., Wareham N.J., Khaw K.-T., Imamura F., Forouhi N.G. (2016). Prospective association of the Mediterranean diet with cardiovascular disease incidence and mortality and its population impact in a non-Mediterranean population: the EPIC-Norfolk study. BMC Med.

[14] The InterAct Consortium (2011). Mediterranean diet and type 2 diabetes risk in the European prospective investigation into cancer and nutrition (EPIC) study. Diabetes Care.

[15] Day N., Oakes S., Luben R., Khaw K.-T., Bingham S., Welch A. (1999). EPIC-Norfolk: study design and characteristics of the cohort. European prospective investigation of cancer. Br. J. Cancer..

[16] Bingham S.A., Gill C., Welch A., Day K., Cassidy A., Khaw K.-T. (1994). Comparison of dietary assessment methods in nutritional epidemiology: weighed records v. 24 h recalls, food-frequency questionnaires and estimated-diet records. Br. J. Nutr..

[17] Bingham S.A., Gill C., Welch A., Cassidy A., Runswick S., Oakes S S. (1997). Validation of dietary assessment methods in the UK arm of EPIC using weighed records, and 24-hour urinary nitrogen and potassium and serum vitamin C and carotenoids as biomarkers. Int. J. Epidemiol..

[18] Bingham S.A., Cassidy A., Cole T.J., Welch A., Runswick S., Black A.E. (1995). Validation of weighed records and other methods of dietary assessment using the 24 h urine nitrogen technique and other biological markers. Br. J. Nutr..

[19] Trichopoulou A., Orfanos P., Norat T., Bueno-de-Mesquita B., Ocké M.C., Peeters P.H. (2005). Modified mediterranean diet and survival: EPIC-elderly prospective cohort study. BMJ.

[20] Bach-Faig A., Berry E.M., Lairon D., Reguant J., Trichopoulou A., Dernini S. (2011). Mediterranean diet pyramid today: Science and cultural updates. Public Health Nutr.

[21] Chamnan P., Simmons R.K., Jackson R., Khaw K.T., Wareham N.J., Griffin S.J. (2011). Non-diabetic hyperglycaemia and cardiovascular risk: moving beyond categorisation to individual interpretation of absolute risk. Diabetologia.

[22] Wareham N.J., Jakes R.W., Rennie K.L., Schuit J., Mitchell J., Hennings S. (2003). Validity and repeatability of a simple index derived from the short physical activity questionnaire used in the European prospective investigation into cancer and nutrition (EPIC) study. Public Health Nutr.

[23] Shohaimi S., Luben R., Wareham N., Day N., Bingham S., Welch A. (2003). Residential area deprivation predicts smoking habit independently of individual educational level and occupational social class: a cross sectional study in the Norfolk cohort of the European Investigation into Cancer (EPIC-Norfolk). J. Epidemiol. Community Health..

[24] McFadden E., Luben R., Wareham N., Bingham S., Khaw K.-T. (2008). Occupational social class, educational level, smoking and body mass index, and cause-specific mortality in men and women: a prospective study in the European prospective investigation of cancer and nutrition in Norfolk (EPIC-Norfolk) cohort. Eur. J. Epidemiol..

[25] Putter H., Fiocco M., Geskus R.B. (2007). Tutorial in biostatistics: competing risks and multi-state models. Stat. Med..

[26] Han Y., Hu Y., Yu C., Guo Y., Pei P., Yang L. (2021). Lifestyle, cardiometabolic disease, and multimorbidity in a prospective Chinese study. Eur. Heart J..

[27] Laffond A., Rivera-Picón C., Rodríguez-Muñoz P.M., Juárez-Vela R., Ruiz De Viñaspre-Hernández R., Navas-Echazarreta N. (2023). Mediterranean diet for primary and secondary prevention of cardiovascular disease and mortality: an updated systematic review. Nutrients.

[28] Mozaffarian D., Marfisi R., Levantesi G., Silletta M.G., Tavazzi L., Tognoni G. (2007). Incidence of new-onset diabetes and impaired fasting glucose in patients with recent myocardial infarction and the effect of clinical and lifestyle risk factors. Lancet.

[29] Kahleova H., Salas-Salvadó J., Rahelić D., Kendall C.W., Rembert E., Sievenpiper J.L. (2019). Dietary patterns and cardiometabolic outcomes in diabetes: a summary of systematic reviews and meta-analyses. Nutrients.

[30] Liu X., Li Y., Ao Y., Zhang L., Zhuang P., Wan X. (2023). Healthy dietary patterns and risk of cardiovascular disease in diabetic patients: a prospective cohort study. Food Funct.

[31] Lind L., Sundström J., Ärnlöv J., Lampa E. (2018). Impact of aging on the strength of cardiovascular risk factors: a longitudinal study over 40 years. J. Am. Heart Assoc..

[32] Lewington S., Whitlock G., Clarke R., Sherliker P., Emberson J., Prospective Studies Collaboration (2007). Blood cholesterol and vascular mortality by age, sex, and blood pressure: a meta-analysis of individual data from 61 prospective studies with 55,000 vascular deaths. Lancet.

[33] Damluji A.A., Forman D.E., Wang T.Y., Chikwe J., Kunadian V., Rich M.W. (2023). Management of acute coronary syndrome in the older adult population: a scientific statement from the American Heart Association. Circulation.

[34] Darmon N., Drewnowski A. (2015). Contribution of food prices and diet cost to socioeconomic disparities in diet quality and health: a systematic review and analysis. Nutr. Rev..

[35] Ross J.S., Bernheim S.M., Bradley E.H., Teng H.-M., Gallo W.T. (2007). Use of preventive care by the working poor in the United States. Prev. Med..

[36] Walker R.J., Gebregziabher M., Martin-Harris B., Egede L.E. (2014). Independent effects of socioeconomic and psychological social determinants of health on self-care and outcomes in Type 2 diabetes, Gen. Hosp. Psychiatry.

[37] Cha E., Choi Y., Bancks M., Faulkner M.S., Dunbar S.B., Umpierrez G.E. (2024). Longitudinal changes in diet quality and food intake before and after diabetes awareness in American adults: the coronary artery risk development in young adults (CARDIA) study. BMJ Open Diabetes Res. Care.

[38] Bray B.D., Paley L., Hoffman A., James M., Gompertz P., Wolfe C.D.A. (2018). Socioeconomic disparities in first stroke incidence, quality of care, and survival: a nationwide registry-based cohort study of 44 million adults in England. Lancet Public Health.

[39] Leung G., Stanner S. (2011). Diets of minority ethnic groups in the UK: influence on chronic disease risk and implications for prevention. Nutr. Bull..

