# Supplementary material for: Prospective Association of the Mediterranean Diet with the Onset of Cardiometabolic Multimorbidity in a UK-Based Cohort: The EPIC-Norfolk Study
Source: J Nutr. 2024 Oct 17;154(12):3761–9. doi: 10.1016/j.tjnut.2024.10.027 (PMC11662236; doi:10.1016/j.tjnut.2024.10.027)
Supplement: Multimedia component 1 [file mmc1.docx]

**Prospective association of the Mediterranean diet with the onset of cardiometabolic multimorbidity in a UK-based cohort: the EPIC-Norfolk study**

Qiaoye Wang, Amand Floriaan, Schmidt, and S. Goya Wannamethee

**Supplementary Material**

Supplementary Table 1

Median-based Mediterranean Diet Score (m-MDS) components and scoring criteria

| **Median-based MDS** | | |
| --- | --- | --- |
| **Component** | **Score = 0** | **Score = 1** |
| Vegetables | Below cohort sex-specific median | Above cohort sex-specific median |
| Fruits and nuts | Below cohort sex-specific median | Above cohort sex-specific median |
| Legumes | Below cohort sex-specific median | Above cohort sex-specific median |
| Cereal | Below cohort sex-specific median | Above cohort sex-specific median |
| Fish | Below cohort sex-specific median | Above cohort sex-specific median |
| Meat | Above cohort sex-specific median | Below cohort sex-specific median |
| Dairy | Above cohort sex-specific median | Below cohort sex-specific median |
| Alcohol | <10 or >50 g/day for men,  <5 or >25 g/day for women | 10 to 50 g/day for men,  5 to 25 g/day for women |
| Ratio of monounsaturated and polyunsaturated fatty acids to saturated fatty acids | Below cohort sex-specific median | Above cohort sex-specific median |

Supplementary Table 2

Pyramid-based Mediterranean Diet Score (pyr-MDS) components and scoring criteria

| **Pyramid-based MDS** | | | |
| --- | --- | --- | --- |
| **Component ^a^** | **Recommended intake** | **Score = 0** | **Score = 1** |
| Vegetables | ≥6/d | 0/d | ≥6/d |
| Legumes | ≥2/wk | 0/wk | ≥2/wk |
| Fruits | 3-6/d | 0/d | 3-6/d |
| Nuts | 1-2/d | 0/d | 1-2/d |
| Cereals | 3-6/d | 0/d | 3-6/d |
| Dairy | 2/d | 0/d | 1.5-2.5/d |
| Fish | ≥2/wk | 0/wk | ≥2/wk |
| Red meat | ˂2/wk | ≥4/wk | ˂2/wk |
| Processed meat | ≤1/wk | ≥2/wk | ≤1/wk |
| White meat | 2/wk | 0/wk | 1.5-2.5/wk |
| Egg | 2-4/wk | 0/wk | 2-4/wk |
| Potato | ≤3/wk | ≥6/wk | ≤3/wk |
| Sweets | ≤2/wk | ≥4/wk | ≤2/wk |
| Alcohol ^b^ | 2/d for men,  1/d for women | ≥4/d for men,  ≥2/d for women | 1.5-2.5/d for men,  0.5-1.5/d for women |
| Olive oil ^c^ | Principal source of  dietary lipids | Non-consumers | Consumers |

^a.^ For these components for which a high consumption was recommended (vegetables, legumes, and fish), continuous scores from 0 to 1 were assigned proportionally from no consumption to meeting the recommended level of consumption.

For components for which moderate consumption was recommended (Fruits, nuts, cereals, dairy, white meat, and egg), a score of 1 was assigned for consumption within the recommendation levels and 0 for no consumption, with consumption levels in between scored proportionately. Overconsumption (double the mid-point value of the recommended intake) was penalized and received a maximum score of 0.5, with consumption between the recommended level and the penalty point scored proportionally.

For these components for which a low consumption was recommended (Red meat, processed meat, potato, and sweets), consumption below the recommended levels was assigned a score of 1 and double the recommended levels were assigned a score of 0, with levels in between scored proportionally.
^b.^ For alcohol a score of 1 for consumption levels within recommendation. Non-consumption was scored 0.5 while overconsumption was scored 0.
^c^*^.^* For olive oil all non-consumers were scored 0 and all consumers 1.

Supplementary Figure 1


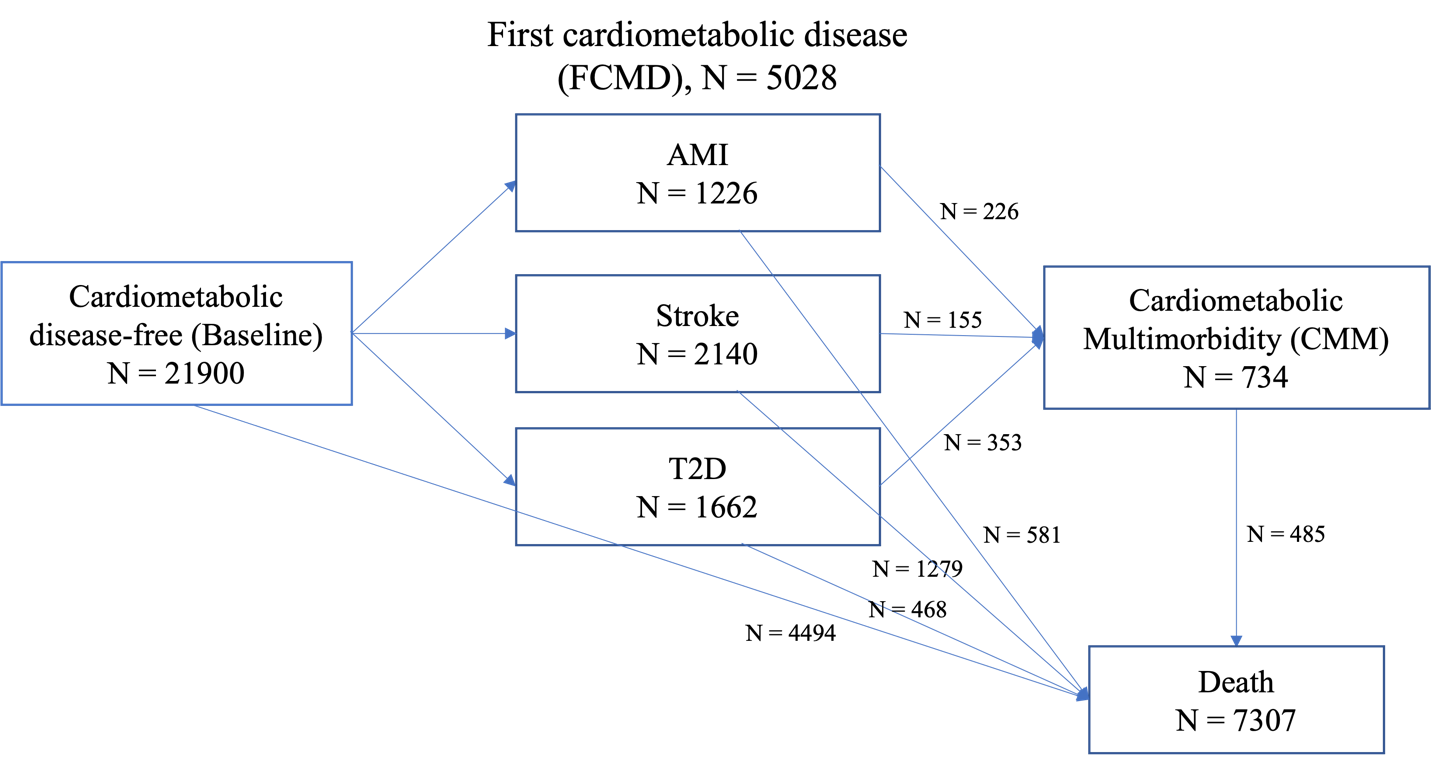


AMI: Acute myocardial infarction, T2D: type 2 diabetes.

Supplementary Table 3

Comparison of complete cases and missing samples, among EPIC-Norfolk participants free of prevalence cardiometabolic disease at baseline in 1993-1997 (N=27885)

| **Variable** | **Complete cases**  **(N = 21900)** | **Missing sample**  **(N = 5985)** | **P-value** |
| --- | --- | --- | --- |
| CMM events, n (%) | 734 (3.4) | 284 (4.8) | < 0.01 |
| Male, n (%) | 9631 (44.0) | 2497 (41.7) | < 0.01 |
| Age at baseline, years | 58.6 (9.2) | 59.7 (10.0) | < 0.01 |
| Body mass index, kg/m^2^ | 26.2 (3.8) | N = 1521  26.8 (4.2) | < 0.01 |
| Median-based MDS  Quartile 1, n (%) | 7277 (33.2) | N = 784  289 (36.9) | 0.2 |
| Pyramid-based MDS  Quartile 1, n (%) | 5479 (25.0) | N = 784  211 (26.9) | 0.1 |
| Education level  Bachelor or above, n (%) | 2890 (13.2) | N = 5985  589 (9.8) | < 0.01 |
| Marital status  Married, n (%) | 18067 (82.5) | N = 5841  4248 (72.7) | < 0.01 |
| Smoking status  Current smokers, n (%) | 2560 (11.7) | N = 5755  922 (16.0) | < 0.01 |
| Physical activity level  Inactive, n (%) | 6262 (28.6) | N = 5984  2435 (40.7) | < 0.01 |
| Social class  Non-manual workers, n (%) | 13281 (60.6) | N = 5324  2710 (50.9) | < 0.01 |
| Family history of MI, n (%) | 7937 (36.2) | N = 5980  1937 (32.4) | < 0.01 |
| Family history of stroke, n (%) | 5335 (24.4) | N = 5980  1323 (22.1) | < 0.01 |
| Family history of diabetes, n (%) | 2752 (12.6) | N = 5980  669 (11.2) | < 0.01 |
| Use of anti-hypertensive drug, n (%) | 3479 (15.9) | N = 5985  303 (5.1) | < 0.01 |
| Use of lipid-lowering drugs, n (%) | 227 (1.0) | N = 5985  15 (0.3) | < 0.01 |

MDS: Mediterranean diet score; CMM: Cardiometabolic multimorbidity; MI: Myocardial infarction.

Summary statistics in mean (SD) or frequency (percentage) unless stated otherwise.

Fisher’s exact test was used for all categorical variables.

Wilcoxon test was used for all continuous variables.

Supplementary Table 4

The Hazard Ratios (HRs) of the associations between the Mediterranean diet and risk of CMM in EPIC-Norfolk participants aged 40-79 in 1993-1997 over a median follow-up of 21.4 years, after imputing missing variables.

| **MDSs** |  |
| --- | --- |
| **Median-based MDS (0-9)** | **HR (95%) of CMM** |
| Per SD increase | 0.89 (0.83, 0.96) * |
| Q1 | Ref |
| Q2 | 0.98 (0.83, 1.17) |
| Q3 | 0.78 (0.63, 0.97) * |
| Q4 | 0.78 (0.64, 0.94) * |
|  |  |
| **Pyramid-based MDS (0-15)** | **HR (95%) of CMM** |
| Per SD increase | 0.90 (0.83, 0.97) * |
| Q1 | Ref |
| Q2 | 0.98 (0.82, 1.18) |
| Q3 | 0.80 (0.65, 0.98) * |
| Q4 | 0.84 (0.68, 1.03) |

CMM: Cardiometabolic multimorbidity; CI: Confidence interval; HR: Hazard ratio; MDS: Mediterranean diet score. Each SD unit corresponds to 1.78 m-MDS and 1.32 pyr-MDS points. Model adjusted for age, sex, BMI, smoking status, physical activity, social class, education level, marital status, medication use (anti-hypertensive drugs or lipid-lowering drugs), and family history of MI, stroke, or diabetes.

* p-value < 0.05

Supplementary Table 5

Hazard Ratios (95% CI) for disease transitions from baseline (CMD-free) to first AMI, stroke, T2D, and CMM by m-MDS in EPIC-Norfolk study participants aged 40-79 years in 1993-1997, over the follow-up duration of 10, 15, and a median of 21.4 years (N=21900)

| **Follow-up duration of 10 years** | | **Hazard Ratios (95% CI) for each disease transition** | | | | |
| --- | --- | --- | --- | --- | --- | --- |
|  |  | **Baseline m-MDS** | | | | |
| **Disease transition** | **No. of Events** | **Per SD increase** | **Q1** | **Q2** | **Q3** | **Q4** |
| Baseline to FCMD | |  |  |  |  |  |
| AMI | 538 | 0.94 (0.86, 1.03) | Ref | 1.04 (0.83, 1.30) | 0.93 (0.73, 1.18) | 0.90 (0.71, 1.14) |
| Stroke | 472 | 0.98 (0.89, 1.07) | Ref | 0.91 (0.70, 1.16) | 0.91 (0.70, 1.17) | 0.93 (0.73, 1.18) |
| T2D | 347 | 0.89 (0.80, 1.00) * | Ref | 0.90 (0.68, 1.19) | 0.67 (0.49, 0.92) * | 0.79 (0.59, 1.04) |
| FCMD to CMM | |  |  |  |  |  |
| AMI to CMM | 28 | 0.89 (0.58, 1.39) | Ref | 1.55 (0.56, 4.31) | 2.06 (0.70, 6.05) | 0.50 (0.12, 2.15) |
| Stroke to CMM | 22 | 1.07 (0.69, 1.67) | Ref | 0.80 (1.99, 3.22) | 0.41 (0.08, 2.14) | 1.43 (0.45, 4.50) |
| T2D to CMM | 47 | 0.97 (0.69, 1.37) | Ref | 1.18 (0.53, 2.60) | 1.10 (0.46, 2.65) | 0.77 (0.30, 2.01) |
| **Follow-up duration of 15 years** | | **Hazard Ratios (95% CI) for each disease transition** | | | | |
|  |  | **Baseline m-MDS** | | | | |
| **Disease transition** | **No. of Events** | **Per SD increase** | **Q1** | **Q2** | **Q3** | **Q4** |
| Baseline to FCMD |  |  |  |  |  |  |
| AMI | 819 | 0.93 (0.87, 1.00) | Ref | 1.01 (0.84, 1.22) | 0.92 (0.75, 1.12) | 0.85 (0.71, 1.03) |
| Stroke | 1016 | 0.95 (0.89, 1.01) | Ref | 0.94 (0.79, 1.12) | 0.99 (0.84, 1.18) | 0.87 (0.74, 1.03) |
| T2D | 839 | 0.90 (0.84, 0.96) * | Ref | 0.96 (0.80, 1.15) | 0.77 (0.63, 0.94) * | 0.82 (0.68, 0.98) * |
| FCMD to CMM |  |  |  |  |  |  |
| AMI to CMM | 81 | 0.84 (0.67, 1.06) | Ref | 1.39 (0.79, 2.45) | 1.05 (0.55, 2.02) | 0.64 (0.34, 1.22) |
| Stroke to CMM | 53 | 1.00 (0.76, 1.33) | Ref | 0.83 (0.35, 1.93) | 0.90 (0.41, 1.99) | 1.40 (0.68, 2.89) |
| T2D to CMM | 126 | 0.98 (0.81, 1.19) | Ref | 1.01 (0.64, 1.59) | 1.05 (0.63, 1.76) | 0.88 (0.52, 1.48) |
| **Entire follow-up duration - a median of 21.4 years** | | **Hazard Ratios (95% CI) for each disease transition** | | | | |
|  |  | **Baseline m-MDS** | | | | |
| **Disease transition** | **No. of Events** | **Per SD increase** | **Q1** | **Q2** | **Q3** | **Q4** |
| Baseline to FCMD | |  |  |  |  |  |
| AMI | 1226 | 0.95 (0.89, 1.00) | Ref | 0.96 (0.83, 1.12) | 0.91 (0.78, 1.07) | 0.89 (0.76, 1.03) |
| Stroke | 2140 | 0.95 (0.90, 0.99) * | Ref | 0.92 (0.82, 1.04) | 0.97 (0.86, 1.09) | 0.86 (0.77, 0.97) * |
| T2D | 1662 | 0.90 (0.85, 0.94) * | Ref | 0.93 (0.81, 1.06) | 0.80 (0.70, 0.92) * | 0.82 (0.72, 0.93) * |
| FCMD to CMM | |  |  |  |  |  |
| AMI to CMM | 226 | 0.94 (0.82, 1.08) | Ref | 0.98 (0.68, 1.42) | 0.92 (0.62, 1.37) | 0.83 (0.58, 1.18) |
| Stroke to CMM | 155 | 0.95 (0.81, 1.13) | Ref | 1.03 (0.66, 1.60) | 0.80 (0.50, 1.26) | 1.04 (0.67, 1.61) |
| T2D to CMM | 353 | 0.93 (0.84, 1.04) | Ref | 1.06 (0.81, 1.39) | 0.85 (0.62, 1.17) | 0.77 (0.57, 1.04) |

AMI: Acute myocardial infarction; CMM: Cardiometabolic multimorbidity; m-MDS: median-based Mediterranean diet score; T2D: Type 2 diabetes. Each SD unit corresponds to 1.78 m-MDS points. m-MDS Q1: score=0-3, n=7277; Q2: score=4, n=4512; Q3: score=5, n=4317; Q4: score=6-9, n=5794. Model adjusted for age, sex, BMI, smoking status, physical activity, social class, marital status, education level, medication use (anti-hypertensive drugs or lipid-lowering drugs), and family history of MI, stroke, or diabetes.

* p-value < 0.05

Supplementary Table 6

Hazard Ratios (95% CI) for disease transitions from baseline (CMD-free) to first AMI, stroke, T2D, and CMM by pyr-MDS in EPIC-Norfolk study participants aged 40-79 years in 1993-1997, over the follow-up duration of 10, 15, and a median of 21.4 years (N=21900)

| **Follow-up duration of 10 years** | | **Hazard Ratios (95% CI) for each disease transition** | | | | |
| --- | --- | --- | --- | --- | --- | --- |
|  |  | **Baseline pyr-MDS** | | | | |
| **Disease transition** | **No. of Events** | **Per SD increase** | **Q1** | **Q2** | **Q3** | **Q4** |
| Baseline to FCMD | |  |  |  |  |  |
| AMI | 538 | 0.93 (0.85, 1.02) | Ref | 0.91 (0.73, 1.14) | 0.92 (0.73, 1.16) | 0.78 (0.60, 1.02) |
| Stroke | 472 | 0.89 (0.81, 0.98) * | Ref | 0.78 (0.61, 1.00) * | 0.90 (0.71, 1.15) | 0.70 (0.53, 0.92) * |
| T2D | 347 | 0.92 (0.82, 1.03) | Ref | 0.86 (0.65, 1.14) | 0.82 (0.61, 1.10) | 0.81 (0.59, 1.11) |
| FCMD to CMM | |  |  |  |  |  |
| AMI to CMM | 28 | 0.55 (0.33, 0.91) * | Ref | 0.46 (0.16, 1.33) | 0.37 (0.11, 1.30) | 0.44 (0.12, 1.65) |
| Stroke to CMM | 22 | 0.92 (0.51, 1.34) | Ref | 0.66 (0.17, 2.62) | 0.64 (0.19, 2.11) | 0.48 (0.10, 2.18) |
| T2D to CMM | 47 | 0.68 (0.47, 0.96) * | Ref | 0.64 (0.29, 1.41) | 0.49 (0.20, 1.20) | 0.52 (0.19, 1.41) |
| **Follow-up duration of 15 years** | | **Hazard Ratios (95% CI) for each disease transition** | | | | |
|  |  | **Baseline pyr-MDS** | | | | |
| **Disease transition** | **No. of Events** | **Per SD increase** | **Q1** | **Q2** | **Q3** | **Q4** |
| Baseline to FCMD | |  |  |  |  |  |
| AMI | 819 | 0.91 (0.84, 0.98) * | Ref | 0.84 (0.70, 1.01) | 0.88 (0.73, 1.06) | 0.76 (0.61, 0.94) * |
| Stroke | 1016 | 0.92 (0.86, 0.98) * | Ref | 0.89 (0.76, 1.06) | 0.93 (0.79, 1.10) | 0.78 (0.64, 0.94) * |
| T2D | 839 | 0.90 (0.84, 0.97) * | Ref | 0.89 (0.75, 1.07) | 0.77 (0.64, 0.94) * | 0.81 (0.66, 0.99) * |
| FCMD to CMM | |  |  |  |  |  |
| AMI to CMM | 81 | 0.68 (0.53, 0.87) * | Ref | 0.60 (0.34, 1.08) | 0.36 (0.18, 0.73) * | 0.61 (0.31, 1.17) |
| Stroke to CMM | 53 | 0.85 (0.63, 1.14) | Ref | 0.73 (0.35, 1.52) | 0.48 (0.22, 1.08) | 0.84 (0.38, 1.87) |
| T2D to CMM | 126 | 0.87 (0.71, 1.06) | Ref | 1.07 (0.68, 1.67) | 0.70 (0.41, 1.20) | 0.78 (0.45, 1.34) |
| **Entire follow-up duration - a median of 21.4 years** | | **Hazard Ratios (95% CI) for each disease transition** | | | | |
|  |  | **Baseline pyr-MDS** | | | | |
| **Disease transition** | **No. of Events** | **Per SD increase** | **Q1** | **Q2** | **Q3** | **Q4** |
| Baseline to FCMD | |  |  |  |  |  |
| AMI | 1226 | 0.93 (0.88, 0.99) * | Ref | 0.87 (0.75, 1.01) | 0.88 (0.75, 1.03) | 0.83 (0.70, 0.99) * |
| Stroke | 2140 | 0.93 (0.89, 0.97) * | Ref | 0.91 (0.81, 1.02) | 0.90 (0.80, 1.02) | 0.84 (0.74, 0.95) * |
| T2D | 1662 | 0.93 (0.88, 0.97) * | Ref | 0.98 (0.86, 1.12) | 0.85 (0.74, 0.98) * | 0.84 (0.73, 0.98) * |
| FCMD to CMM | |  |  |  |  |  |
| AMI to CMM | 226 | 0.89 (0.77, 1.03) | Ref | 1.09 (0.76, 1.55) | 0.73 (0.49, 1.09) | 0.80 (0.53, 1.22) |
| Stroke to CMM | 155 | 0.96 (0.81, 1.14) | Ref | 0.76 (0.49, 1.17) | 0.66 (0.42, 1.03) | 1.02 (0.65, 1.60) |
| T2D to CMM | 353 | 0.95 (0.84, 1.06) | Ref | 1.08 (0.80, 1.39) | 0.92 (0.68, 1.25) | 0.89 (0.65, 1.23) |

AMI: Acute myocardial infarction; CMM: Cardiometabolic multimorbidity; pyr-MDS: pyramid-based Mediterranean diet score; T2D: Type 2 diabetes. Each SD unit corresponds to 1.32 pyr-MDS points. pyr-MDS Q1: score=0-7.55, n=5479; Q2: score=7.56-8.45, n=5502; Q3: score=8.46-9.34, n=5465; Q4: score=9.35-15, n=5454. Model adjusted for age, sex, BMI, smoking status, physical activity, marital status, education level, medication use (anti-hypertensive drugs or lipid-lowering drugs), and family history of MI, stroke, or diabetes.

* p-value < 0.05

Supplementary Figure 2

Dose-response curve for the association between m-MDS and pyr-MDS and the risk of CMM.


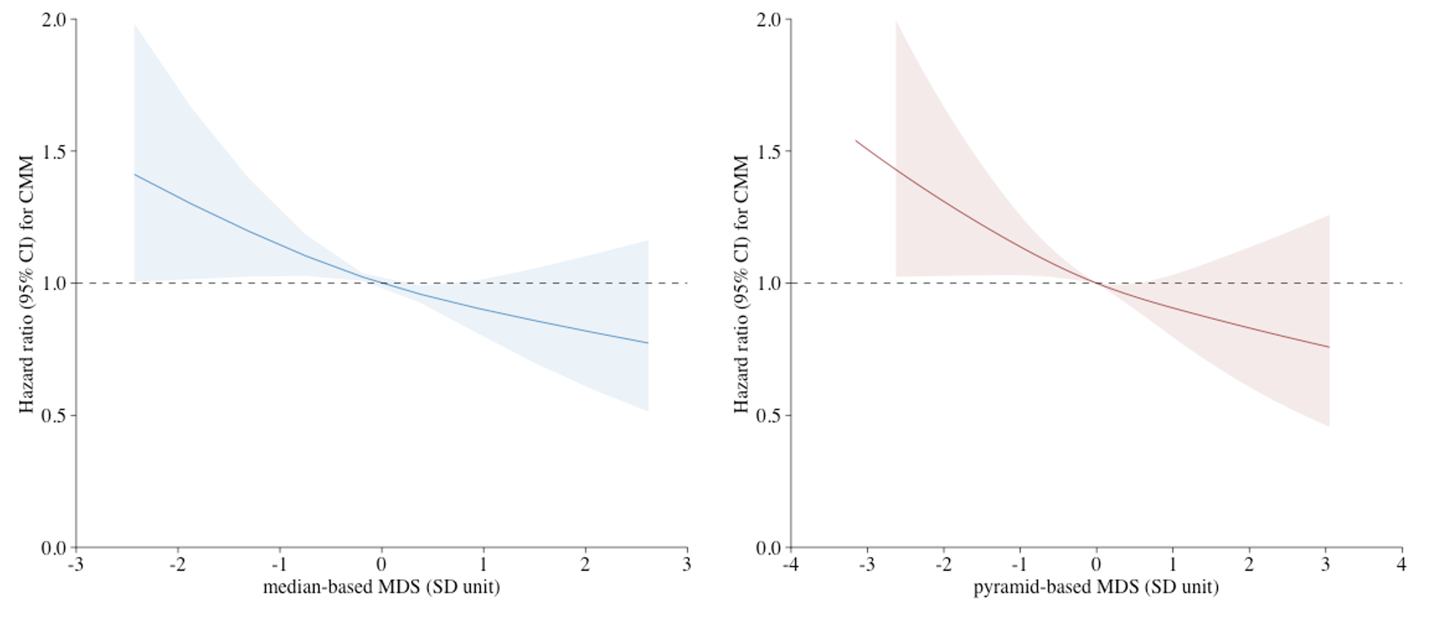


CMM: Cardiometabolic multimorbidity; MDS: Mediterranean diet score; m-MDS: median-based Mediterranean diet score; pyr-MDS: pyramid-based Mediterranean diet score.

Restricted cubic spline curves (knots = 3) were drawn for the associations between m-MDS (left) and pyr-MDS (right) in standard deviation units and the risk of CMM. One standard deviation unit in m-MDS equals to 1.78 points and one standard deviation unit in pyr-MDS equals to 1.32 points. Covariates included in the models were age, sex, BMI, smoking status, physical activity, social class, education level, marital status, medication use (anti-hypertensive drugs or lipid-lowering drugs), and family history of MI, stroke, or diabetes.

Supplementary Table 7

Prospective association between adherence to the Mediterranean diet (measured by the m-MDS and pyr-MDS) and transition risks of CMM over a median follow-up of 21.4 years, excluding BMI from the model covariates (N=21900).

|  |  | **Hazard Ratios (95% CI) for each disease transition** | | | | |
| --- | --- | --- | --- | --- | --- | --- |
|  | | **Baseline m-MDS** | | | | |
| **Disease transition** | **No. of Events** | **Per SD increase** | **Q1** | **Q2** | **Q3** | **Q4** |
| **Baseline to FCMD** | 5028 | 0.93 (0.90, 0.96) * | Ref | 0.93 (0.86, 1.01) | 0.90 (0.83, 0.98) * | 0.85 (0.79, 0.92) * |
| **FCMD to CMM** | 734 | 0.94 (0.87, 1.01) | Ref | 1.04 (0.86, 1.26) | 0.81 (0.65, 1.00) | 0.85 (0.70, 1.04) |
|  | | **Baseline pyr-MDS** | | | | |
| **Disease transition** | **No. of Events** | **Per SD increase** | **Q1** | **Q2** | **Q3** | **Q4** |
| **Baseline to FCMD** | 5028 | 0.93 (0.90, 0.96) * | Ref | 0.93 (0.86, 1.01) | 0.90 (0.83, 0.97) * | 0.83 (0.77, 0.91) * |
| **FCMD to CMM** | 734 | 0.94 (0.87, 1.01) | Ref | 1.02 (0.85, 1.24) | 0.82 (0.67, 1.01) | 0.91 (0.73, 1.13) |

FCMD: First cardiometabolic disease; CMM: Cardiometabolic multimorbidity; m-MDS: median-based Mediterranean diet score; pyr-MDS: pyramid-based Mediterranean diet score.

Each SD unit corresponds to 1.78 m-MDS and 1.32 pyr-MDS points. m-MDS Q1: score=0-3, n=7277; Q2: score=4, n=4512; Q3: score=5, n=4317; Q4: score=6-9, n=5794. pyr-MDS Q1: score=0-7.55, n=5479; Q2: score=7.56-8.45, n=5502; Q3: score=8.46-9.34, n=5465; Q4: score=9.35-15, n=5454. Model adjusted for age, sex, smoking status, physical activity, social class, education level, marital status, medication use (anti-hypertensive drugs or lipid-lowering drugs), and family history of MI, stroke, or diabetes.

* p-value < 0.05

Supplementary Table 8

Hazard Ratios (95% CI) for disease transition risks of CMM by pyramid-based MDS (pyr-MDS) in EPIC-Norfolk study aged 40-79 years in 1993-1997 over a median follow-up of 21.4 years, stratified by age (N=21900)

|  |  | **Hazard Ratios (95% CI) for each disease transition** | | | | |
| --- | --- | --- | --- | --- | --- | --- |
| **Younger participants (< 60 years old)**  **(n = 12165)** | | **Baseline pyr-MDS** | | | | |
| **Disease transition** | **No. of Events** | **Per SD increase** | **Q1** | **Q2** | **Q3** | **Q4** |
| **Baseline to FCMD** | 1704 | 0.91 (0.86, 0.95) * | Ref | 0.93 (0.81, 1.05) | 0.87 (0.76, 0.99) * | 0.78 (0.68, 0.90) * |
| **FCMD to CMM** | 204 | 0.97 (0.83, 1.13) | Ref | 0.95 (0.66, 1.36) | 0.87 (0.58, 1.29) | 0.98 (0.63, 1.52) |
|  |  |  |  |  |  |  |
| **Older participants (**$\boldsymbol{\geq}$ **60 years old)**  **(n = 9735)** | | **Baseline pyr-MDS** | | | | |
| **Disease transition** | **No. of Events** | **Per SD increase** | **Q1** | **Q2** | **Q3** | **Q4** |
| **Baseline to FCMD** | 3324 | 0.95 (0.91, 0.98) * | Ref | 0.93 (0.84, 1.02) | 0.89 (0.81, 0.98) * | 0.87 (0.79, 0.97) * |
| **FCMD to CMM** | 530 | 0.93 (0.84, 1.02) | Ref | 1.06 (0.85, 1.33) | 0.83 (0.65, 1.05) | 0.89 (0.69, 1.15) |

P-value for interaction = 0.1.

AMI: Acute myocardial infarction; FCMD: First cardiometabolic disease; CMM: Cardiometabolic multimorbidity; pyr-MDS: pyramid-based Mediterranean diet score. Each SD unit corresponds to 1.32 pyr-MDS points.

pyr-MDS Q1: score=0-7.55, N for younger participants=2923, N for older participants=2556; Q2: score=7.56-8.45, N for younger participants=2931, N for older participants=2571; Q3: score=8.46-9.34, N for younger participants=3051, N for older participants=2414; Q4: score=9.35-15, N for younger participants=3260, N for older participants=2194.

Model adjusted for age, sex, BMI, smoking status, physical activity, social class, marital status, education level, medication use (anti-hypertensive drugs or lipid-lowering drugs), and family history of MI, stroke, or diabetes.

* p-value < 0.05

Supplementary Table 9

Hazard Ratios (95% CI) for disease transitions risks of CMM by pyramid-based MDS (pyr-MDS) in EPIC-Norfolk study aged 40-79 years in 1993-1997 over a median follow-up of 21.4 years, stratified by sex (N=21900)

|  |  | **Hazard Ratios (95% CI) for each disease transition** | | | | |
| --- | --- | --- | --- | --- | --- | --- |
| **Male participants**  **(n = 9631)** | | **Baseline pyr-MDS** | | | | |
| **Disease transition** | **No. of Events** | **Per SD increase** | **Q1** | **Q2** | **Q3** | **Q4** |
| **Baseline to FCMD** | 2575 | 0.91 (0.87, 0.95) * | Ref | 0.92 (0.83, 1.02) | 0.87 (0.78, 0.96) * | 0.81 (0.72, 0.92) * |
| **FCMD to CMM** | 408 | 0.94 (0.85, 1.04) | Ref | 0.96 (0.75, 1.23) | 0.88 (0.67, 1.16) | 0.94 (0.69, 1.28) |
|  |  |  |  |  |  |  |
| **Female participants**  **(n = 12269)** | | **Baseline pyr-MDS** | | | | |
| **Disease transition** | **No. of Events** | **Per SD increase** | **Q1** | **Q2** | **Q3** | **Q4** |
| **Baseline to FCMD** | 2453 | 0.95 (0.91, 0.99) * | Ref | 0.94 (0.83, 1.05) | 0.90 (0.80, 1.01) | 0.86 (0.76, 0.97) * |
| **FCMD to CMM** | 326 | 0.94 (0.83, 1.06) | Ref | 1.11 (0.82, 1.51) | 0.77 (0.56, 1.07) | 0.91 (0.65, 1.25) |

P-value for interaction = 0.1.

AMI: Acute myocardial infarction; FCMD: First cardiometabolic disease; CMM: Cardiometabolic multimorbidity; pyr-MDS: pyramid-based Mediterranean diet score. Each SD unit corresponds to 1.32 pyr-MDS points.

pyr-MDS Q1: score=0-7.55, N for male participants=3191, N for female participants=2288; Q2: score=7.56-8.45, N for male participants=2602, N for female participants=2900; Q3: score=8.46-9.34, N for male participants=2196, N for female participants=3269; Q4: score=9.35-15, N for male participants=1642, N for female participants=3812.

Model adjusted for age, BMI, smoking status, physical activity, social class, marital status, education level, medication use (anti-hypertensive drugs or lipid-lowering drugs), and family history of MI, stroke, or diabetes.

* p-value < 0.05

Supplementary Table 10

Hazard Ratios (95% CI) for disease transitions from baseline (CMD-free) to FCMD and CMM by low or high adherence to the pyr-MDS in EPIC-Norfolk study aged 40-79 years in 1993-1997 over a median follow-up of 21.4 years (N=21900)

|  |  | **Hazard Ratios (95% CI) for each disease transition** |
| --- | --- | --- |
| **Low adherence to the Mediterranean diet**  **(pyr-MDS** $\boldsymbol{\leq}$ **8.45, n = 10,981)** | | **Baseline pyr-MDS** |
| **Disease transition** | **No. of Events** | **Per SD increase** |
| Baseline to FCMD | 2771 | 0.95 (0.92, 0.99) * |
| FCMD to CMM | 433 | 0.97 (0.88, 1.06) |
| **High adherence to the Mediterranean diet**  **(pyr-MDS > 8.45, n = 10,919)** | | **Baseline pyr-MDS** |
| **Disease transition** | **No. of Events** | **Per SD increase** |
| Baseline to FCMD | 2257 | 0.96 (0.92, 1.00) |
| FCMD to CMM | 301 | 1.04 (0.92, 1.18) |

FCMD: First cardiometabolic disease; CMM: Cardiometabolic multimorbidity; pyr-MDS: pyramid-based Mediterranean diet score. Each SD unit corresponds to 0.79 pyr-MDS points. Model adjusted for age, sex, BMI, smoking status, physical activity, marital status, education level, medication use (anti-hypertensive drugs or lipid-lowering drugs), and family history of MI, stroke, or diabetes.

* p-value < 0.05
